# Spatial Selective Choroidal Stromal and Vascular Changes in Intermediate AMD: Insights From High-Density Optical Coherence Tomography Analysis

**DOI:** 10.1167/iovs.67.5.49

**Published:** 2026-05-19

**Authors:** Meenakshi Kumar, Matt Trinh, Rupesh Agrawal, Lisa Nivison-Smith

**Affiliations:** 1 School of Optometry and Vision Science, University of New South Wales, Sydney, Australia; 2 Centre for Eye Health, University of New South Wales, Sydney, Australia; 3 National Healthcare Group Eye Institute, Tan Tock Seng Hospital, Singapore, Singapore; 4 Lee Kong Chian School of Medicine, Nanyang Technological University, Singapore, Singapore; 5 Singapore Eye Research Institute, Singapore, Singapore; 6 Eye ACP Program, Duke NUS Medical School, Singapore, Singapore

**Keywords:** choroidal vascularity index, choroidal angioarchitecture, iAMD, parafovea, rod susceptibility

## Abstract

**Purpose:**

To characterize spatially selective alterations in choroidal angioarchitecture in intermediate AMD (iAMD) using high-density optical coherence tomography (OCT) analysis.

**Methods:**

This retrospective study analysed Spectralis OCT macular cube scans (∼61 B-scans) from 83 eyes with iAMD and 83 age-matched controls. A customized Python pipeline derived from ‘Choroidalyzer’ was developed to compute choroidal thickness (CT), the choroidal vascularity index (CVI), lumen area (LA), and stromal area (SA) across a 60 × 60 section of macula, yielding ∼3600 grid-level metrics. Spatial variations were evaluated using Early Treatment Diabetic Retinopathy Study sectors, and eccentricity-based modelling.

**Results:**

There were 83 eyes in each group with no difference in baseline characteristics (*P* = 0.135–1.00). Global analysis revealed significant reductions for CVI (−1.57%) and LA (−2.43%) in iAMD (both *P* < 0.0001). Spatial analysis indicated significant distinctions between outer and near-periphery rings of the Early Treatment Diabetic Retinopathy Study template (*P* = 0.0001–0.0096) and was supported by eccentricity analysis that showed a subfoveal increase (CVI, + 18.21%; LA, +17.77%; *P* < 0.0001) and parafoveal loss in iAMD eyes (CVI, −13.68 %, LA, −22.82%; *P* < 0.0001). Spatial analysis of CT showed linear increase with eccentricity (*y* = 1.16*x* − 1.79; *R*^2^ = 0.19; *P* < 0.001) with thinning subfoveally. SA demonstrated a similar pattern of loss (*y* = −2.081*x*^2^ + 8.72*x* – 8.14; *R*^2^ = 0.553; *P* < 0.001).

**Conclusions:**

Choroidal metrics in iAMD exhibit spatially selective changes: CT showed central thinning with minimal peripheral alterations, whereas CVI displayed subfoveal increase and parafoveal reduction. SA and LA changes mimicked CT and CVI, suggesting they drive thickness and vascularity changes, respectively. These findings suggest that the parafoveal selectivity observed in early iAMD may be secondary to choroidal vascular deficits and highlight the value of high-density OCT for detecting subtle remodeling.

Age related macular degeneration (AMD) is a multifactorial, progressive eye disease and a leading cause of vision loss worldwide, with its burden expected to increase in parallel with population ageing.^1^^–^^6^ Despite extensive research, the mechanisms underlying early disease onset and progression remain incompletely understood. Increasing evidence implicates choroidal dysfunction in AMD pathogenesis due to its role in retinal metabolism. Postmortem analyses of eyes with drusen demonstrate elevated inflammatory markers and complement dysregulation in comparison with non-AMD eyes.^7^^–^^9^ In vivo imaging studies similarly report reduced choroidal thickness (CT), focal choriocapillaris dropout, and zones of nonperfusion, even at early disease stages.^10^^–^^12^ Optical coherence tomography (OCT) angiography has further revealed age- and drusen-related increases in choriocapillaris flow deficits, and histology confirms sclerosed choriocapillaris, hyalinized Bruch's membrane, and basal laminar deposits.^5^^,^^13^^–^^16^

Based on these observations, it is understood that there is a need to measure choroidal changes clinically in early stages as well. Current findings around choroidal metrics in intermediate AMD (iAMD) are conflicting, perhaps because of the limited consideration for the choroid's natural topographical variation^17^^,^^18^ or conflicting results.^19^^,^^20^ For example, the choroidal vascularity index (CVI), a measure of choroidal angioarchitecture using OCT B-scans,^21^^,^^22^ follows a concentric pattern, with higher values at the fovea as well as a superior/inferior bias.^23^ However, studies investigating the CVI in exudative and nonexudative AMD have mostly been used on coarse spatial templates that do not consistently match this topography, risking statistical bias and masking biologically meaningful regional patterns.^24^^,^^25^

Although global and sectoral choroidal measurements provide useful information, they fail to capture the fine spatial heterogeneity in early AMD. For example, imaging studies have shown focal choroidal loss alongside AMD lesions such as localized choriocapillaris flow deficits in regions underlying reticular pseudodrusen.^26^ Additionally, given that rods exhibit selective parafoveal vulnerability in early AMD^27^^–^^29^ preceding foveal cone involvement, there is a strong rationale for spatially resolved analysis that can align structural vascular changes with functional.^30^

To address, these gaps, we developed a high-density spatial OCT analysis capable of high-density assessment. Prior applications of this approach have revealed spatially patterned loss across individual retinal layers in AMD, offering insight into disease-specific pathoanatomy.^31^^,^^32^ In this study, we apply this high-density topographical method to eyes with iAMD to characterize spatially selective alterations in choroidal structure and vascularity. By delineating regional vulnerabilities in choroid, our findings aim to provide new mechanistic insights into the role of the choroid in early AMD.

## Methods

### Study Population

This retrospective study included patients who attended the Centre for Eye Health (CFEH), Sydney, Australia, between July 2010 and July 2022. CFEH is a referral-based clinic providing advanced ocular diagnostics by optometrists and ophthalmologists.^33^

The clinical diagnosis of iAMD was based on the Beckman classification^34^ using fundus photography due to availability in retrospective data and access to OCT images for confirmation of classification. The healthy control group included participants and volunteers who were referred to the CFEH and found to have no evidence of any posterior segment diseases based on ocular examination, including OCT and mydriatic fundus photography. Participants diagnosed with iAMD presenting with large drusen (>125 µm in diameter) or pigmentary abnormalities relating to AMD with medium drusen (63–125 µm in diameter) were included as the case group. Eyes with other posterior segment diseases or with signs of late AMD (macular neovascularization, geographical atrophy, or disciform scarring) were excluded from this study. Eligibility for both groups of participants was age older than 50 years; visual acuity of 20/25 or better; a spherical equivalent of less than 6 diopters and astigmatism of less than 3 diopters; an IOP of less than 21 mm Hg; no systemic vascular-related disease like hypertension, diabetes mellitus, or cardiovascular abnormalities; no mental or cognitive impairment based on self-reported medical history; and no history of smoking. Participants were a subset of a propensity-matched samples additionally screened for nonsmokers and absent systemic vascular diseases^31^^,^^32^ specifically for choroidal assessments. All participants in this study gave informed consent for their deidentified data to be used in research in accordance with the tenets of the Declaration of Helsinki, and the study was approved by the Biomedical Human Research Ethics Advisory Panel of the University of New South Wales.

### Imaging Protocol

All eligible participants had a volume scan with Spectralis SD-OCT, which were macular cube scans consisting of 61 B-scans (scan length, 8.6 mm; scan depth, 1.8 mm; axial resolution, 3.9 microns) covering an area of 30° × 25°^35^ (Heidelberg Engineering, Heidelberg, Germany). From this, a single, randomly selected eye from each participant was extracted in control group and the affected eye in the iAMD group. If a participant had multiple scans in their file, the latest macular cube scan was used. All scans with a quality score of less than 15 dB or poor visibility of choroid due to opacities or shadowing were excluded. If three or more B-scans of the macular cube were to be excluded from a participant for quality reasons, then the participant was excluded from this study. No participants were eliminated for these criteria.

### Choroidal Metrics

**Figure 1.**
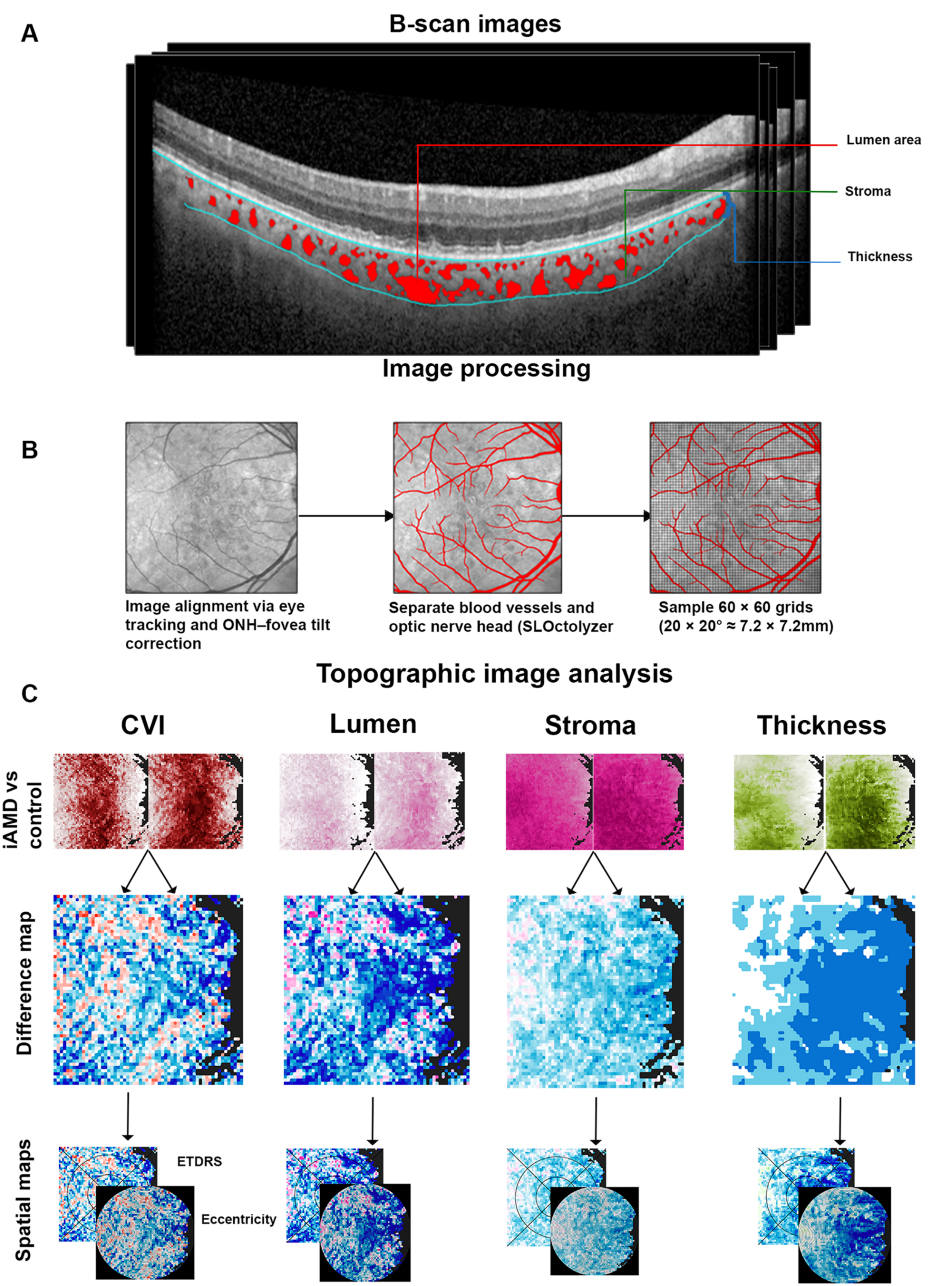
Calculation of choroidal metric across macula based on the pipeline described by Englemann and Burke. (**A**) Process of identification of lumen and stroma within the choroid, with CT measurement. (B) Removal of overlying blood vessels to enhance segmentation process. (C) Generation of topographic map for all choroidal metrics for baseline and follow-up. These maps were then used to generate a difference map, which were then subjected to spatial templates.

A customized pipeline was developed in Python derived from OCTolyzer and Choroidalyzer^36^^,^^37^ to calculate choroidal parameters. CT was defined as layer from the RPE to choroidoscleral boundary.^37^ The CVI and area measurements were built upon methodology described by Agrawal et al.^21^ for luminal area (LA), stromal area (SA), and CVI as a ratio of LA to the total choroidal area (LA + SA); however, thresholding was replaced by multiscale median cut quantization instead of Niblack's threshold.^37^ A global analysis was initially conducted in the absence of spatial templates, that is, the median of the entire macular scan area. These measurements were then calculated in a spatially resolved manner by dividing the macular cube into a 60 × 60 grid, covering an area of 7200 × 7200 micrometers (corresponding with 25° × 25°). This grid-based approach ensured comprehensive coverage of all 61 B-scans within the volume (Fig. 1).

### Image Processing

A macular volume scan of selected participants was extracted in .VOL and .XML files. The images were then processed through a modified OCTolyzer program.^36^ This program segments and excludes blood vessels and the optic nerve head imaging across all B-scans, hence mitigating the effect of shadowing on choroidal imaging. The pixel intensity was then standardized using the formula ($\frac{iAMD-healthy\;control}{iAMD}*100$) to account for asymmetry in rate of change and further adjusted using the formula ($\frac{iAMD-healthy\;controls}{\left[\frac{iAMD+healthy\;controls}{2}\right]}$ *100 ) to account for unbounded maximum values wherein the increases could approach infinity if the baseline (denominator) was near zero.^38^ This methodology was applied to all the grids and visualized as topographical maps.

### Spatial Analysis

The 60 × 60 grids were interpreted with two templates to analyze spatial changes, namely, the following:

1. An Early Treatment Diabetic Retinopathy Study (ETDRS) template with 1-mm, 3-mm, and 6-mm diameter concentric rings and regions outside this considered near-peripheral region^39^^,^^40^; and
2. An eccentricity analysis with 0.05-mm increments to match grid resolution.

### Statistical Analyses

Statistical analyses were performed using python 3.12.6 (Python Software Foundation, Beaverton, OR, USA) and visualized using GraphPad prism 10.0.3 (GraphPad, Boston, MA, USA). A *P* value of 0.05 was considered significant. Normality assumptions were checked using Shapiro Wilks test, and median [interquartile range] were used across all the measures and graphs. Global comparisons were done using Wilcoxon sign-ranked *t*-test. Eccentricity vs. choroidal parameters were assessed using linear/quadratic regression; *R*^2^ was used to determine appropriate fit.

## Results

### Study Population

Eighty-three eyes with iAMD from 83 participants and 83 normal eyes were included in this study. The demographic details of the participants are provided in Table 1. The study and control group was significantly different in ethnicity, best-corrected visual acuity, and fellow eye status (*P* < 0.000–0.001). All covariables were adjusted for during the analysis.

**Table 1.** Demographic Details of the Study Population

	Control (*N* = 83)	iAMD (*N* = 83)	*P* Value
Demographic/health features			
Age (years)	65.01	67.28	0.135^*^
Sex (male:female) (%)	43:57	43:57	1.000**^†^**
Ethnicity (White:Asian:other:unknown) (%)	68:16:14:2	46:20:6:28	**<0.000** **^†^**
Neurological disorders (i.e., migraine present) (%)	53	54	0.876**^†^**
Spherical equivalent refraction (diopters)	0.63	0.48	0.926^*^
Best-corrected visual acuity (logMAR)	0.00	0.10	**<0.001** ^*^
AMD-specific features			
Fellow eye status (normal:mixedAMD:other) (%)	100:0:0	18:78:4	**<0.001** **^†^**
Pigmentary abnormalities (present) (%)	–	33	–
Reticular pseudodrusen (present) (%)	–	23	–
Drusen load (mm^3^)	–	0.004 ± 0.031	–

All continuous data variables are presented as mean ± SD (Range) and categorical variables as percentage of eyes. Presence of metabolic disorders included neurological diseases such as migraines. Boldface entries indicate statistical significance.

* *t*-test.

^†^ χ^2^ test.

### Global Analysis

Global analysis found no significant change in CT (*P* = 0.41, Wilcoxon test) or SA (*P* = 0.48, Wilcoxon test) between control eyes and those with iAMD. Global CVI and LA, however, showed a statistically significant decrease in iAMD vs. control eyes (CVI, −1.57% [IQR, −3.67 to 1.07], *P* < 0.01; LA, −2.43% [IQR, −5.33 to 0.00], *P* < 0.05, Wilcoxon test).

### Spatial Analysis

**Figure 2.**
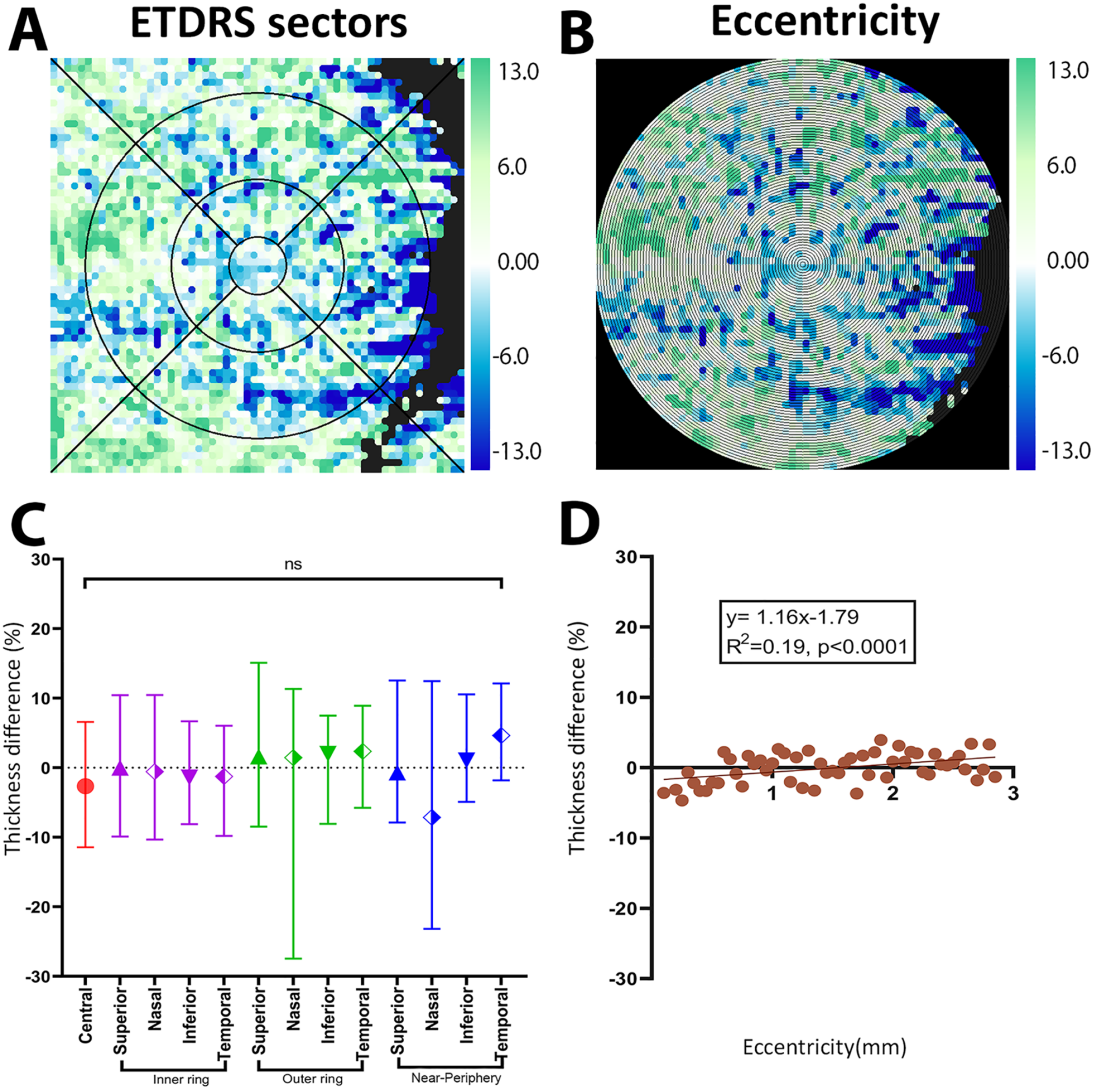
CT difference maps between iAMD and controls. (**A**, **C**) ETDRS sector analysis indicating no significant difference between iAMD eyes and control in any sector. (**B**, **D**) Eccentricity analysis indicating significant linear increase in CT from fovea to near periphery.

Changes to choroidal parameters between iAMD and normal eyes were then visualized spatially by dividing the global macular area to 60 × 60 equal grids and generating spatial topography maps (Fig. 2A–B). Standard spatial templates (ETDRS template, eccentricity) were then applied and differences between controls and iAMD eyes reassessed.

### CT

As with the global analysis, no significant differences in CT were observed between iAMD eyes compared with normal eyes within the ETDRS sectors (*P* > 0.05 for all sectors) (Figs. 2A, 2C, Supplementary Table S1). Across eccentricities from 0.10 to 2.85 mm, CT showed a modest relation with greatest loss in iAMD eyes near the fovea −4.67%, followed by a linear increase crossing over at 1.5 mm toward the near-peripheral region (*y* = 1.16*x* − 1.79; *R*^2^ = 0.19; *P* < 0.0001) (Figs. 2B, 2D).

### CVI

**Figure 3.**
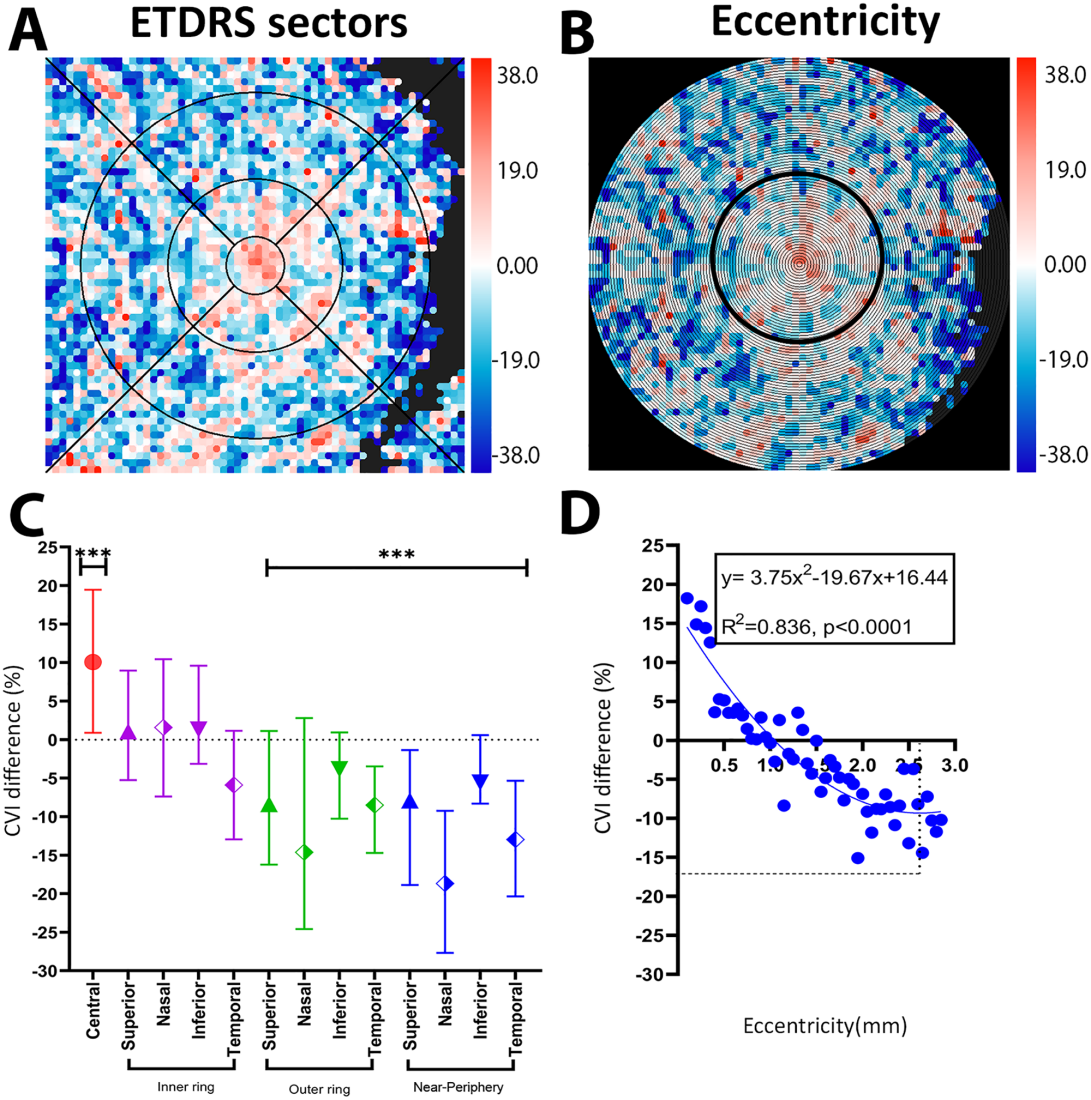
CVI difference map between the iAMD and control groups. (**A**, **C**) ETDRS sector analysis and scatter plot, indicating some significant sectoral distinctions. (**B**, **D**) Eccentricity analysis indicating a quadratic function with highest increase at fovea and loss starting at 1.0 mm increasing to 2.6 mm, then stabilizing. ***P < 0.0001.

Unlike CT, some ETDRS sectoral differences were observed in the CVI between iAMD eyes vs. control eyes. Centrally, the CVI was increased by 10.08% [IQR, −6.57 to 28.68] in iAMD, whereas the outer and near-peripheral rings showed significant reductions (Figs. 3A, 3C) (Kruskal–Wallis test, *P* < 0.05) (Supplementary Table S1). Modeled as a function of eccentricity, the CVI followed a quadratic pattern (*y* = 3.75*x*^2^ − 19.67*x* + 16.44; *R*^2^ = 0.83; *P* < 0.0001) (Figs. 3B, 3D), with a central increase in iAMD of up to 18.22%, and a rapid decrease toward the parafovea (1 mm) to a maximal loss of −13.71% at 2.5 mm. This loss in CVI then reduced in slope/magnitude approaching 3 mm.

### Choroidal Area Measurements (Lumen Area [LA] and Stroma Area [SA])

**Figure 4.**
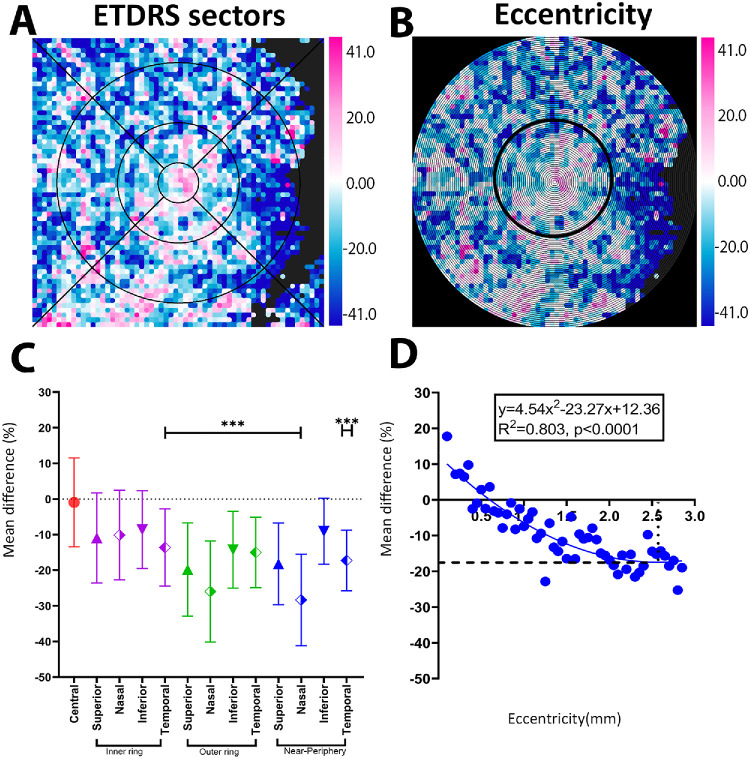
LA difference map between iAMD and control group. (**A**, **C**) ETDRS analysis and scatter plot, indicating some significant sectoral distinctions. (**B**, **D**) Eccentricity analysis with quadratic function from 0 mm to 3 mm, indicating the highest increase at fovea and loss starting at 0.50 mm, and maximum loss at 2.25 mm, then stabilizing similar to CVI. ***P < 0.0001.

To contextualize differences in the CVI, we further examined its constituents, that is, the choroidal LA and SA. LA difference maps showed mixed regions of thinning and thickening similar to the CVI (Figs. 4A, 4B). This variation was also observed in the ETDRS sector analysis, where the inner temporal, all outer ring sectors, and all near-peripheral sectors except the inferior quadrant showed statistically significant reductions in the LA in iAMD eyes compared with controls (Kruskal–Wallis test, *P* = 0.012–0.0001) (Figs. 4A,  4C, Supplementary Table S1). An eccentricity analysis also showed that LA exhibited a quadratic pattern of change, with greater LA in iAMD eyes centrally (at 0 mm eccentricity; 17.77%) and a decrease toward the parafovea with greatest loss of −21.56% at 2.3 mm, then stabilizing as approaching the near-peripheral region (LA, *y* = 4.54*x*^2^ − 23.27*x* + 12.36; *R*^2^ = 0.803; *P* < 0.0001) (Figs. 4B, 4D).

**Figure 5.**
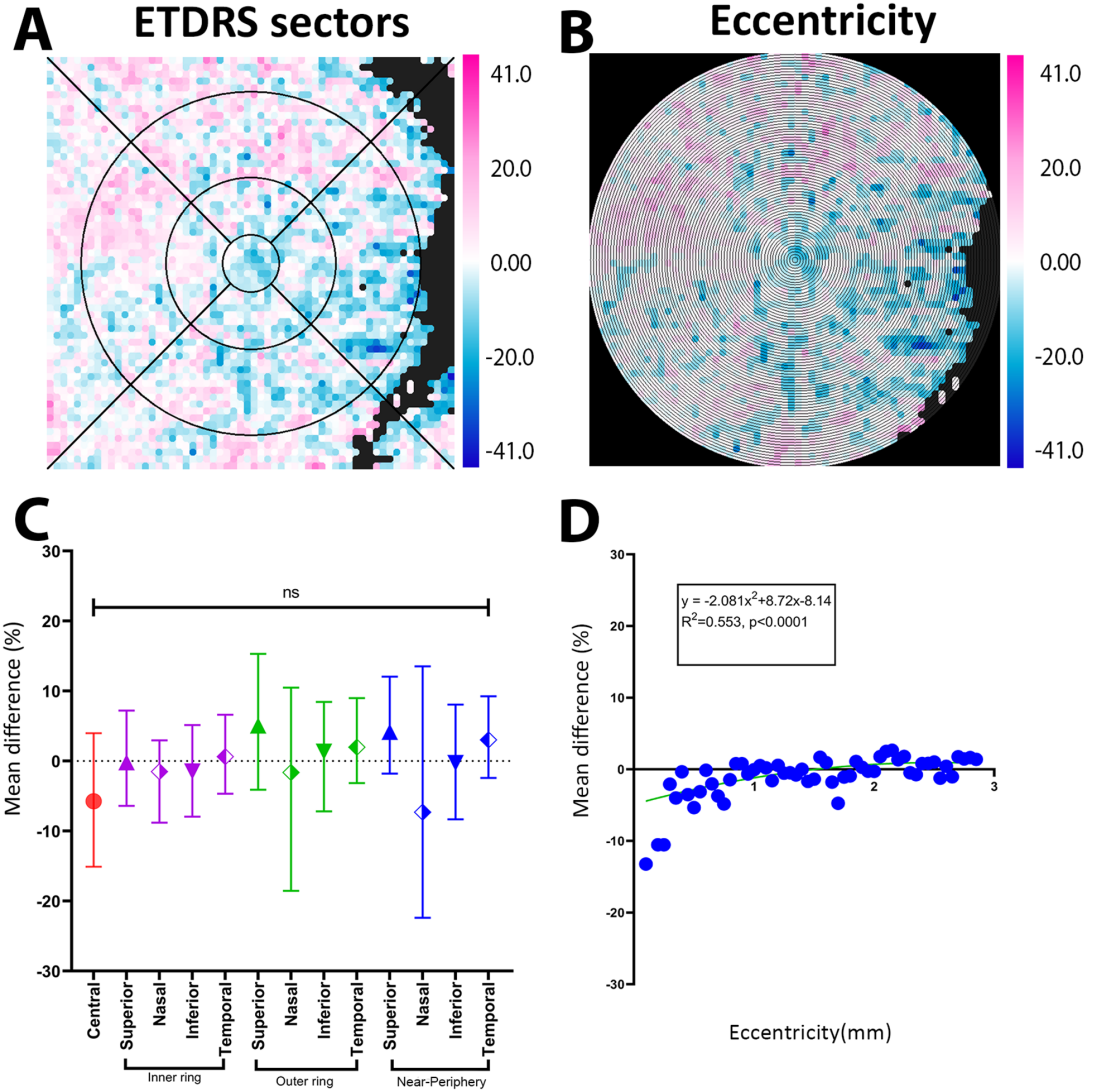
SA difference map between iAMD and control group. (**A**, **D**) ETDRS analysis and scatter plot, showing no significant difference. (**B**, **E**) Eccentricity analysis with a quadratic function from 0 to 3.0 mm with the greatest loss at fovea then crossing over to increased SA at 1.5 mm, finally plateauing at the near periphery region. ***P < 0.0001.

Last, SA topographical difference maps also showed mixed regions of thinning and thickening (Fig. 5). Spatial analyses, however, were similar to CT findings, with no significant differences within the ETDRS sector analysis (Figs. 5A, 5C, Supplementary Table S1); an eccentricity analysis showed a quadratic decline with greatest loss at the fovea (−13.22%), transitioning over to a modest increase from 1.5 mm eccentricity (*y* = −2.08*x*^2^ + 8.72*x* − 8.14; *R*^2^ = 0.55; *P* < 0.0001) (Figs. 5B, 5D).

### Correlation of Choroidal Metrics

In view of the spatial similarities between the CVI and the LA and the CT and the SA, a Spearman correlation analysis was performed. For interpretability, eccentricity data were presented.

Both the CVI and LA and CT and SA showed a statistically significant correlation (CVI:LA, *r* = 0.834; CT:SA, *r* = 0.50; *P* < 0.0001) (Supplementary Fig. S1), suggesting that CVI changes stem from changes to vasculature, whereas thickness changes stem partly from changes to the stroma in AMD.

## Discussion

This study found that the choroid undergoes distinct spatial patterns of structural and vascular changes in iAMD. Specifically, the CVI was increased centrally and decreased parafoveally, whereas CT showed a mild subfoveal reduction that was minimized with eccentricity in iAMD eyes compared with age-matched controls. LA changes appear to drive CVI and the SA demonstrated patterns mirroring thickness, suggesting that these components may drive the observed changes. Importantly, global analyses did not capture these variations, particularly for subtle parameters such as CT and SA, whereas the CVI and LA revealed consistent alterations, reinforcing the importance of spatially resolved approaches to understanding choroidal changes in early AMD.

### Spatial and Covariable Adjusted Analyses Reveal Mild Central Choroidal Thinning in iAMD

Several studies have reported conflicting findings regarding CT in iAMD, with some indicating thinning^41^^–^^43^ and others showing no association.^44^^–^^49^ Manjunath et al,^50^ found that CT correlated strongly with age in dry but not wet AMD, suggesting that the thickness change in iAMD may not be primarily disease driven. ALSTAR2 (Alabama Study on Early Age-Related Macular Degeneration 2) reported no differences in the CT between AMD eyes and controls after adjusting for covariates.^48^ Similarly in our study, participants were matched for key covariables, which may explain the absence of global or ETDRS differences.

Spatial analyses, however, revealed localized changes, indicating loss at the subfovea and an increment outside of 1.5 mm, highlighting that, in addition to addressing confounders, the spatial selectivity of the disease process must also be accounted for when assessing CT. In this study, when eccentricity was considered, the maximum loss in CT was at the fovea^51^^,^^52^ and a gradual increase was observed away from the fovea. Confocal and histological studies indicate that the subfovea is the thickest part of the choroid owing to the exceptional metabolic activity of the overlying photoreceptors, making it highly vulnerable. The thinning experienced at the subfovea could be explained by the convergence of multiple mechanisms involving choriocapillaris loss, RPE dysfunction, drusen accumulation, and inflammation at fovea.^10^^,^^52^^,^^53^ However, the magnitude of thinning observed is close to the axial resolution of Spectralis OCT (3.9 µm) and only marginally higher than age-related decline, suggesting that, although statistically significant, CT may lack sensitivity as a biomarker of early AMD, consistent with previous reports,^54^ but can be useful for the study of choroidal pathogenesis in the disease.

### CVI Varies Significantly Between the Subfovea and Parafovea in iAMD

Unlike CT, the CVI displayed a distinct spatial pattern with a subfoveal increase and parafoveal loss. All spatial analyses in this study pointed to a highly varied pattern of change for CVI in iAMD with an increase at the fovea followed by loss pattern around 1 mm eccentricity or the parafovea.^55^ Vascular loss in the parafovea corresponds closely with regions of greatest rod vulnerability, where early psychophysical and functional impairments in scotopic sensitivity, dark adaptation, and contrast perception have been well documented in AMD.^27^^–^^30^ The greater magnitude and spatially distinct nature of CVI changes to CT suggest that the choroidal angioarchitecture in iAMD may undergo significant changes without a significant impact on overall structure. CVI changes, particularly the subfoveal increase with parafoveal hypoperfusion, also significantly differ from normal age-related topographical changes,^23^ suggesting this choroidal remodeling is related to the disease process rather than age,^15^^,^^56^ preceding and potentially driving early functional decline.

A comparison with prior CVI studies is challenging, because most analyses have focused mostly on the subfovea despite evidence that both the healthy choroid^20^^,^^23^^,^^57^ and AMD pathology exhibit sectoral variation.^28^^,^^32^^,^^55^^,^^58^^,^^59^ Literature reports are inconsistent, describing a decrease,^17^^,^^47^^,^^60^ increase,^42^^,^^61^ or no change in the CVI in iAMD.^62^^–^^64^ Longitudinal assessments suggest a biphasic process with the initial increase in the CVI during drusen formation, followed by a reduction, possibly occurring secondary to VEGF expression and initial vascular enlargement due to inflammation.^5^^,^^65^ This dynamic may explain discrepancies in cross-sectional studies, which may have been performed at different time points in this biphasic process.^5^^,^^42^

### LA Appears to Drive the CVI, Whereas SA Drives CT Changes 

The LA showed a similar spatial pattern of change to the CVI, whereas the SA mimicked the pattern of central loss seen in CT. This finding suggests that the LA drives the CVI, and CT originates from SA changes. Previous work has pointed to changes to CT emerging predominantly from the stromal regions.^66^^–^^68^ Meanwhile, the role of the LA is more complicated because the correlation may reflect the use of the LA to calculate the CVI (where the LA is the numerator of the CVI ratio and part of the denominator, which combines the LA and SA to determine total area). Other studies, however, have described choroid shrinkage in AMD, which is found to be greater in the vasculature than the stroma,^17^^,^^54^ suggesting that the LA is affected more. The foveal increase in the LA and CVI (reflected as the engorgement of vessels) observed in this study could be a compensatory act secondary to perfusion deficits arising from choriocapillaris in iAMD.^61^ Parafoveal selectivity is a well established feature in early AMD owing to rod susceptibility in early disease.^28^^,^^55^^,^^58^ A decrease in the LA (and subsequently in the CVI) at the parafovea may relate to the presence of the choroidal watershed zone, which is known to have reduced perfusion owing to vessel termination^69^ and be susceptible to ischemic changes.^57^^,^^67^ Trinh et al.^32^ also demonstrate a parafoveal pattern of loss in the outer retina in iAMD, and it can be argued that the current study substantiates the theory of the choroidal origin of AMD as the primary blood supplier for the outer retina.

Furthermore, previous studies based on laser speckle flowgraphy studies show that, despite an overall reduced blood flow in AMD, choroidal flow seems to be impaired predominantly, reinforcing the causal linkage between vascular compromise and photoreceptor vulnerability in early disease stages.^70^^,^^71^

Clinically, these findings highlight that high-density OCT mapping could serve as a sensitive imaging biomarker for early vascular dysfunction in AMD. The spatial organization of the choroidal vasculature is intrinsically linked to retinal metabolic requirements. By enabling spatially resolved quantification of choroidal remodeling, this approach may help to stratify patients at greater risk of progression to late AMD, complementing conventional risk indicators such as drusen volume or pigmentary change. The integration of choroidal vascular metrics, particularly parafoveal CVI and LA alterations, into clinical assessment protocols could enhance early diagnosis, monitoring of subclinical disease, and timely initiation of preventive interventions.

### Strengths and Limitations

This study's strengths include demonstrating the feasibility of an automated, analytical pipeline developed in Python, derived from Choroidalyzer and OCTolyzer. Beyond its immediate research application, this framework is scalable for large datasets and could be readily adapted for artificial intelligence–based risk prediction or real-time clinical decision support systems.

This study's limitations were its cross-sectional design and population, obtained from a clinical setting, meaning that some physiological factors such as presence of metabolic diseases were ascertained from self-report and, therefore, may not have been as reliable as objective measurement. We were also limited in the availability of ocular parameters and thus could not account for individual differences in axial length. However, we mitigated this issue by including a narrow spectrum of normative refractive error in our study (mean, ±0.68 diopters) and focusing on the use of metrics such as the CVI, which is a ratio designed to mitigate interindividual differences, such as the effect of ocular magnification.

## Future Directions and Conclusions

Future high-density studies with longitudinal design and cross-validation with functional assessments such as microperimetry or dark adaptometry are required to provide structure function concordance to these spatial choroidal signatures.

This study demonstrates that choroidal remodeling in iAMD is pattern specific and was captured using spatial analyses. The use of an automated pipeline further highlights the feasibility of large-scale, reproducible quantification of choroidal architecture.

## Supplementary Material

Supplement 1
