# Supplementary material for: Spatial Selective Choroidal Stromal and Vascular Changes in Intermediate AMD: Insights From High-Density Optical Coherence Tomography Analysis
Source: Invest Ophthalmol Vis Sci. 2026 May 19;67(5):49. doi: 10.1167/iovs.67.5.49 (PMC13206713; doi:10.1167/iovs.67.5.49)

Supplementary Table 1. ETDRS sector values for all choroidal metrics

Grouped sectors	Sectors	Choroidal Thickness Median difference (%)	p value	Choroidal vascularity index Median difference (%)	p value	Lumen Area Median difference (%)	p value	SA Median difference (%)	p value
**Central (1mm)**	Central	-2.64 [-10.12, 4.83]	0.6757	10.08 [2.82, 17.34]	**0.0096**	4.18 [-8.28, 16.63]	0.6192	-5.74 [-12.13, 0.66]	0.1012
**Inner ring(1-3mm)**	Inner-Sup	0.02 [-7.94, 7.98]	0.7958	1.07 [-6.07, 8.22]	0.863	-3.49 [-16.14, 9.16]	0.191	-0.17 [-6.53, 6.19]	0.9349
	Inner-Nas	-0.55 [-8.30, 7.19]	0.7097	1.59 [-5.25, 8.42]	0.8523	-8.34 [-20.90, 4.21]	0.1805	-1.51 [-7.86, 4.83]	0.6207
	Inner-Inf	-1.38 [-8.17, 5.40]	0.824	1.33 [-4.97, 7.63]	0.9746	1.86 [-9.06, 12.77]	0.4593	-1.49 [-7.08, 4.09]	0.6336
	Inner-Temp	-1.24 [-8.00, 5.52]	0.9819	-5.90 [-11.95, 0.16]	0.0509	-10.77 [-21.57, 0.03]	**0.0415**	0.64 [-5.12, 6.40]	0.6433
**Outer ring(3-6mm)**	Outer-Sup	1.62 [-6.75, 9.98]	0.7853	-8.37 [-15.45, -1.29]	**0.0055**	-16.19 [-29.29, -3.10]	**0.012**	5.08 [-1.79, 11.94]	0.2203
	Outer-Nas	1.47 [-7.98, 10.93]	0.1271	-14.61 [-23.17, -6.06]	**0.0042**	-25.30 [-39.53, -11.07]	**0.0011**	-1.63 [-9.77, 6.51]	0.2186
	Outer-Inf	2.01 [-4.56, 8.58]	0.8452	-3.84 [-9.90, 2.23]	**0.0112**	-8.78 [-19.57, 2.00]	**0.0355**	1.34 [-4.00, 6.69]	0.7403
	Outer-Temp	2.36 [-3.67, 8.40]	0.555	-8.50 [-14.42, -2.58]	**0.0006**	-12.87 [-22.73, -3.00]	**0.0077**	1.98 [-3.38, 7.34]	0.3473
**Extramacular(>6mm)**	Extra-Sup	-0.67 [-8.35, 7.00]	0.5162	-7.88 [-13.78, -1.98]	**0.0003**	-13.54 [-25.04, -2.05]	**0.0055**	4.17 [-2.25, 10.60]	0.188
	Extra-Nas	-7.14 [-16.48, 2.21]	0.1463	-18.68 [-27.56, -9.80]	**0.000**	-28.79 [-41.64, -15.93]	**0.0001**	-7.31 [-15.80, 1.18]	0.2433
	Extra-Inf	1.07 [-4.58, 6.72]	0.376	-5.56 [-10.86, -0.26]	**0.0167**	-6.31 [-15.57, 2.95]	0.1746	-0.27 [-5.42, 4.89]	0.7029
	Extra-Temp	4.65 [-0.99, 10.29]	0.3358	-12.97 [-18.91, -7.03]	**0.0001**	-18.41 [-26.93, -9.89]	**0.0004**	3.04 [-2.16, 8.23]	0.4732

Supplementary figure 1.

Correlation matrix between a)Lumen area and CVI and b) Choroidal thickness and stromal area

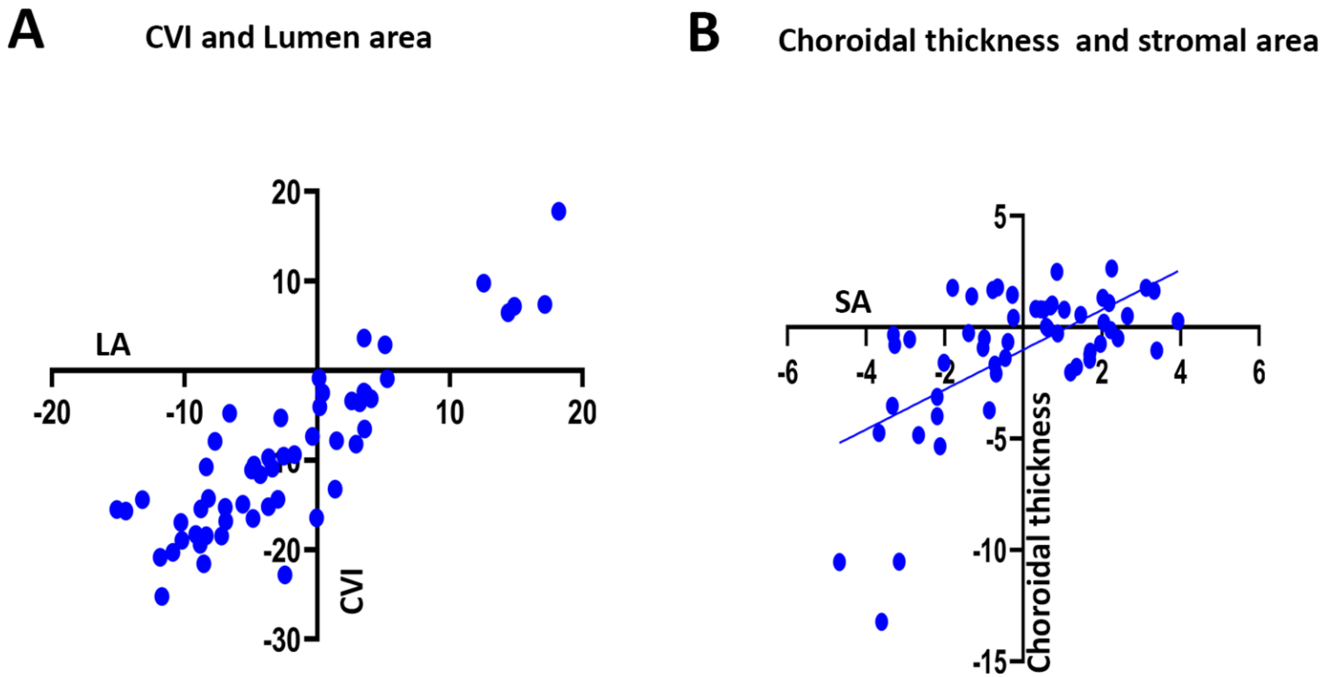

Supplement: Supplement 1 [file iovs-67-5-49_s001.pdf]
